# Exogenous expression of the glycosyltransferase LARGE1 restores α-dystroglycan matriglycan and laminin binding in rhabdomyosarcoma

**DOI:** 10.1186/s13395-019-0195-0

**Published:** 2019-05-04

**Authors:** Daniel Beltrán, Mary E. Anderson, Narendra Bharathy, Teagan P. Settelmeyer, Matthew N. Svalina, Zia Bajwa, John F. Shern, Sakir H. Gultekin, Marco A. Cuellar, Takahiro Yonekawa, Charles Keller, Kevin P. Campbell

**Affiliations:** 1 Department of Molecular Physiology and Biophysics, Department of Neurology, Howard Hughes Medical Institute, University of Iowa Roy J. and Lucille A. Carver College of Medicine, 4283 Carver Biomedical Research Building, 285 Newton Road, Iowa City, IA 52242-1101 USA; 2 Children’s Cancer Therapy Development Institute, 12655 SW Beaverdam Road W, Beaverton, OR 97005 USA; 3 Pediatric Oncology Branch, Center for Cancer Research, National Institutes of Health, Bethesda, MD 20892 USA; 4 Department of Pathology, Oregon Health & Science University, Portland, OR 97239 USA

**Keywords:** Dystroglycan, Matriglycan, *LARGE1*, Rhabdomyosarcoma, Laminin

## Abstract

**Background:**

α-Dystroglycan is the highly glycosylated component of the dystrophin-glycoprotein complex (DGC) that binds with high-affinity to extracellular matrix (ECM) proteins containing laminin-G-like (LG) domains via a unique heteropolysaccharide [-GlcA-beta1,3-Xyl-alpha1,3-]_n_ called matriglycan. Changes in expression of components of the DGC or in the O-glycosylation of α-dystroglycan result in muscular dystrophy but are also observed in certain cancers. In mice, the loss of either of two DGC proteins, dystrophin or α-sarcoglycan, is associated with a high incidence of rhabdomyosarcoma (RMS). In addition, glycosylation of α-dystroglycan is aberrant in a small cohort of human patients with RMS. Since both the glycosylation of α-dystroglycan and its function as an ECM receptor require over 18 post-translational processing enzymes, we hypothesized that understanding its role in the pathogenesis of RMS requires a complete analysis of the expression of dystroglycan-modifying enzymes and the characterization of α-dystroglycan glycosylation in the context of RMS.

**Methods:**

A series of cell lines and biopsy samples from human and mouse RMS were analyzed for the glycosylation status of α-dystroglycan and for expression of the genes encoding the responsible enzymes, in particular those required for the addition of matriglycan. Furthermore, the glycosyltransferase *LARGE1* was ectopically expressed in RMS cells to determine its effects on matriglycan modifications and the ability of α-dystroglycan to function as a laminin receptor.

**Results:**

Immunohistochemistry and immunoblotting of a collection of primary RMS tumors show that although α-dystroglycan is consistently expressed and glycosylated in these tumors, α-dystroglycan lacks matriglycan and the ability to bind laminin. Similarly, in a series of cell lines derived from human and mouse RMS, α-dystroglycan lacks matriglycan modification and the ability to bind laminin. RNAseq data from RMS cell lines was analyzed for expression of the genes known to be involved in α-dystroglycan glycosylation, which revealed that, for most cell lines, the lack of matriglycan can be attributed to the downregulation of the dystroglycan-modifying enzyme *LARGE1*. Ectopic expression of *LARGE1* in these cell cultures restored matriglycan to levels comparable to those in muscle and restored high-affinity laminin binding to α-dystroglycan.

**Conclusions:**

Collectively, our findings demonstrate that a lack of matriglycan on α-dystroglycan is a common feature in RMS due to the downregulation of *LARGE1*, and that ectopic expression of *LARGE1* can restore matriglycan modifications and the ability of α-dystroglycan to function as an ECM receptor.

## Background

Dystroglycan is a ubiquitously expressed extracellular matrix (ECM) receptor and plays key roles in the formation and maintenance of many tissues [1]. In skeletal muscle, dystroglycan is part of the dystrophin-glycoprotein complex (DGC), which establishes a continuous link between the laminin-G-like (LG) domain-containing ECM proteins and the intracellular cytoskeleton. The key role that the DGC plays in muscle function is highlighted by the fact that mutations in almost any gene encoding one of its components can cause muscular dystrophy [2]. Furthermore, mutations in a single component of the DGC can result in instability and loss of the entire complex, highlighting the requirement for the complex to be intact.

Similarly, loss of dystroglycan function has been associated with human pathologies, including muscular dystrophy with or without various degrees of brain and eye defects [1]. Dystroglycan is synthesized as a single precursor that is subsequently cleaved into two subunits that remain tightly but non-covalently linked: the cell surface-associated α-dystroglycan and the membrane-spanning β-dystroglycan. α-Dystroglycan functions as a receptor for several LG domain-containing basement membrane proteins, including laminin [3–5], perlecan [6], agrin [7], and Slit [8], whereas β-dystroglycan binds to proteins involved in cytoskeleton organization (dystrophin and utrophin) [9–11]. α-Dystroglycan requires extensive glycosylation and post-translational processing in order to bind to its extracellular ligands. This functional glycosylation consists of the unique heteropolysaccharide [-GlcA-beta1,3-Xyl-alpha1,3-]_n_ called matriglycan, which has been reported only on α-dystroglycan and binds with high-affinity to ECM proteins that contain LG domains. In humans, mutations in any of the genes coding for the glycosyltransferases involved in this process can lead to a spectrum of syndromes known as secondary dystroglycanopathies. These conditions are characterized by an absence or reduction in the matriglycan modification on α-dystroglycan and by muscular dystrophy with or without brain and eye defects.

We and others have demonstrated that glycosylation of α-dystroglycan is also compromised in a wide variety of cancers, and that this defect correlates with poor prognosis [12–16]. An analysis of knockout mouse models for two of the DGC components, dystrophin (*mdx*) and α-sarcoglycan, demonstrated that primary defects in the DGC are sometimes associated with the formation of rhabdomyosarcomas (RMS) [17]. Specifically, among aged (> 1 year old) mice deficient for dystrophin (*mdx*) or α-sarcoglycan, 21% (32/150) and 5% (4/80) of mice developed such tumors, respectively. Mdx-derived tumors display both cellular and molecular characteristics of embryonal rhabdomyosarcoma (eRMS) and almost no characteristics of alveolar rhabdomyosarcoma (aRMS).

Biochemical evidence supporting the involvement of α-dystroglycan in RMS comes from a manuscript reporting on five RMS tumors, which found the expression of α-dystroglycan to be reduced or absent while that of β-dystroglycan was normal [18]. Parallel studies of human RMS have indicated that dystrophin may have roles as a tumor suppressor [19].

In the study described here, we expand on the above-described investigations by describing a functional role for dystroglycan in RMS, and begin to elucidate the molecular mechanism underlying its altered function. Analysis of a microarray panel containing eRMS and aRMS tissue revealed that matriglycan levels on α-dystroglycan are reduced in both sample types. Similarly, analysis of cell lines derived from human and mouse RMS shows that in a subset of these cell lines, α-dystroglycan lacks matriglycan, and that this is accompanied by reduced expression of the glycosyltransferase LARGE1. Lastly, we found that ectopic expression of *LARGE1* in RMS cells restored both the matriglycan modification of α-dystroglycan and the ability of α-dystroglycan to bind laminin, demonstrating that the loss of *LARGE1* expression causes the lack of matriglycan and a loss of high-affinity laminin binding in RMS.

## Methods

### Experimental replicates

All experiments were repeated in the laboratory three times. Data reported are representative.

### Cell culture

Primary cells from genetically engineered mouse aRMS samples were cultured as previously described [20, 21]. The collection of tumors and the culture of primary cells from human RMS were performed under an IRB approved protocol from Oregon Health & Science University. In brief, for each culture, the tumor was minced and digested with collagenase (10 mg/ml) overnight at 4 °C. The dissociated cells were then incubated in Dulbecco’s modified eagle’s media (DMEM) supplemented with 10% fetal bovine serum (FBS) and 1% penicillin-streptomycin in 5% CO_2_ at 37 °C. Experiments were performed at or before passage 10.

### Adenovirus-mediated gene transfer

The human genes that code for dystroglycan, *POMT1*, *POMT2*, *POMGNT1*, *POMGNT2*, *B3GALNT2*, *POMK*, *LARGE1*, *LARGE2*, *B3GNT1*, *Fukutin*, *FKRP*, *ISPD*, and *TMEM5* were cloned into replication-deficient adeno-viral (Ad5) vectors. Adenovirus was generated by the Viral Vector Core at the University of Iowa. The generation of Ad5 constructs was described previously [5]. Cancer cells were grown to 70–80% confluency in 48-well or 10 cm plates, and were infected with Ad5 constructs at an MOI of 3 in the corresponding complete medium 48 h after infection.

### cDNA synthesis and real-time PCR

Total RNA was extracted from cells in culture using the RNeasy isolation kit (Qiagen). First-strand complementary DNA (cDNA) was synthesized from total RNA using the AMV reverse transcriptase (Roche) and a combination of random hexamers and polyA primers, according to the manufacturer’s instructions. Each of the target genes was amplified in real-time from cDNA using oligonucleotides specific to that gene (sequences and conditions available upon request), with 28S-RNA, Rpl27, and Rpl4 used as the normalization controls. cDNA levels were determined using SYBR green in a MyiQ rt-PCR detection system (BioRad). All samples were run in triplicate.

### Western blotting

Proteins were extracted from cultured cells using lysis buffer (50 mM Tris pH 7.6, 150 mM NaCl, 1% Triton, and protease inhibitor cocktail in 1x PBS). Insoluble material was removed by centrifugation at 12,000×*g* for 15 min. The solubilized supernatant was added to 200 μl of wheat germ agglutinin (WGA) slurry (Vector Labs) and the samples were rotated overnight at 4 °C. The pelleted WGA beads were washed three times with 1 ml TBS containing 0.1% Triton X-100. After the final wash, 250 μl of loading buffer (loading sample buffer (LSB)) was added to the beads. The samples were heated to 99 °C for 10 min before loading into 3–15% SDS–PAGE gels, and protein was subsequently transferred to polyvinylidene fluoride (PVDF) membranes, which were subsequently blocked in 2% nonfat dried milk in TBST buffer (50 mM Tris pH 8.0, 150 mM NaCl, 0.05% Tween 20). All antibodies were diluted in the same buffer that was used for blocking. Membranes were washed in TBST buffer. Blots were developed with IR-conjugated secondary antibodies (Pierce Biotechnology, Rockford, IL) and scanned using an Odyssey infrared imaging system (LI-COR Bioscience, Lincoln, NE). α-Dystroglycan and β-dystroglycan core proteins were detected using antibody AF6868, and matriglycan was detected using antibody IIH6 [22, 23]. Electrophoresis was performed by standard SDS-PAGE methods. Precision Plus Protein™ (BioRad) was used as the molecular weight marker for Western blot analysis.

### Laminin overlay assay

Ligand overlay assays were performed on membranes using mouse Engelbreth–Holm–Swarm (EHS) laminin (Sigma-Aldrich) [24]. Briefly, PVDF membranes were blocked in laminin binding buffer (LBB: 10 mM triethanolamine, 140 mM NaCl, 1 mM MgCl_2_, 1 mM CaCl_2_, pH 7.6) containing 3% bovine serum albumin (BSA) followed by incubation with laminin overnight at 4 °C in LBB. Membranes were washed and incubated with anti-laminin antibody (L9393, Sigma-Aldrich, 1:100) followed by Licor dye-conjugated donkey anti-rabbit IgG.

### Solid-phase assay

WGA eluates were diluted 1:50 in TBS and coated on polystyrene ELISA microplates (Costar) overnight at 4 °C. Plates were washed in LBB and blocked for 1 h in 3% BSA in LBB. Mouse EHS laminin was diluted in LBB and applied for 2 h. Wells were washed with 3% BSA in LBB and incubated for 30 min with anti-laminin antibody (L9393, Sigma-Aldrich, 1:5000 dilution) followed by HRP-conjugated anti-rabbit IgG (Invitrogen, 1:5000 dilution). Plates were developed with o-phenylenediamine dihydrochloride and H_2_O_2_, and then reactions were stopped with 2 N H_2_SO_4_. Absorbance per well was read at 490 nm by a microplate reader.

## Results

A previous study showed a loss of fully glycosylated α-dystroglycan in the majority of RMS samples from a small cohort; notably, the expression of β-dystroglycan, with which α-dystroglycan is co-translated, was not significantly altered [18]. Since the publication of that work in 2007, our understanding of the intricate glycosylation and post-translational processing events required to synthesize the functional form of α-dystroglycan has progressed significantly. In order to validate these results and expand the glycobiological analysis, we performed Western blotting using two distinct antibodies that recognize α-dystroglycan: “anti-core dystroglycan,” a polyclonal antibody that has specificity for epitopes along the backbones of both α-dystroglycan and β-dystroglycan, and thus does not depend on glycosylation status; and monoclonal antibody IIH6 (anti-matriglycan), which binds α-dystroglycan only when it has been modified with matriglycan by LARGE [25]. The Western blot results are complemented by a laminin overlay assay, which measures the ability of α-dystroglycan to bind laminin with high-affinity.

**Fig. 1:**
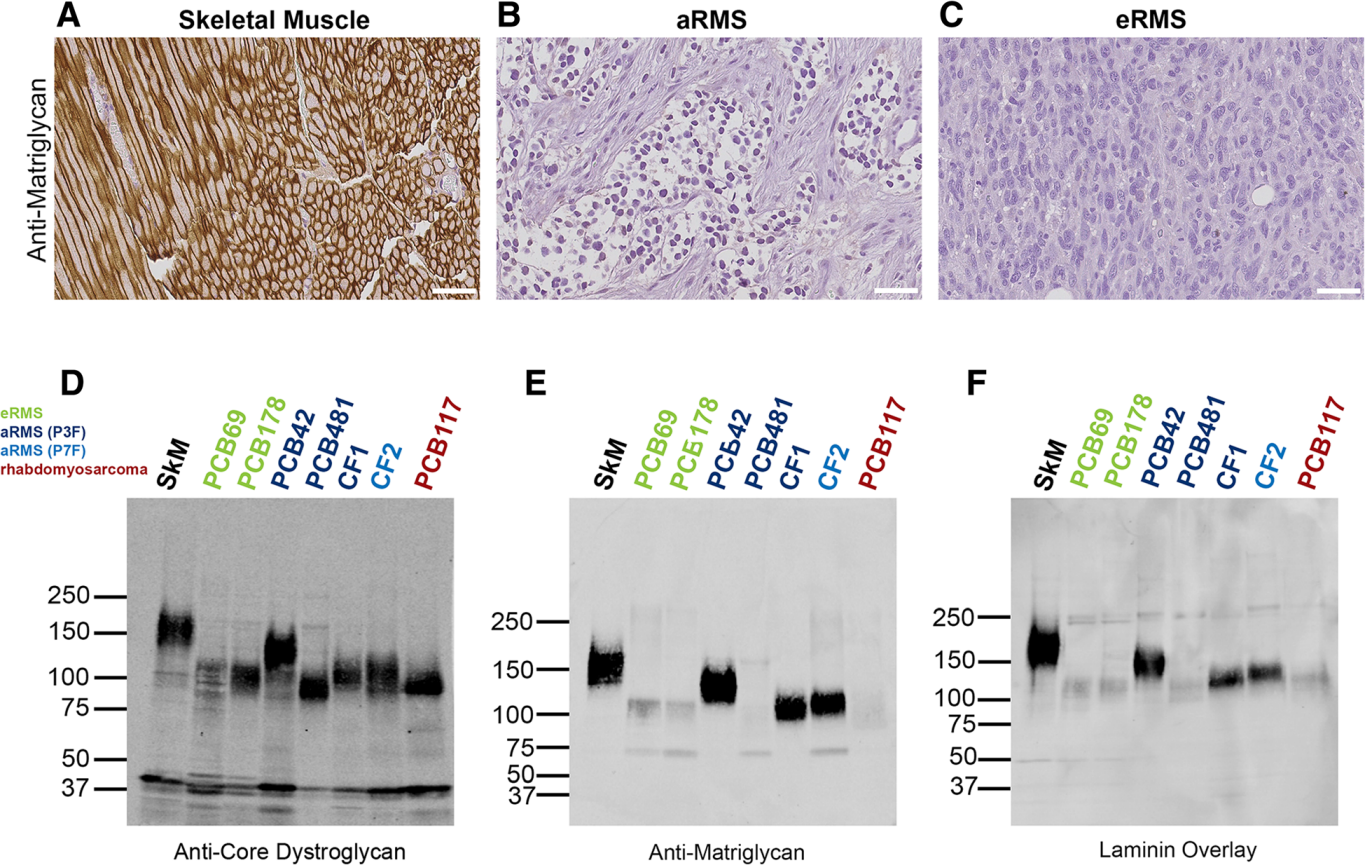
Immunohistochemical staining for α-dystroglycan matriglycan in skeletal muscle and RMS. **a**–**c** Representative images of immunohistochemistry for matriglycan (antibody IIH6) staining of **a** control human skeletal muscle, **b** aRMS, and **c** eRMS. *n* = 55 aRMS, *n* = 14 eRMS. **d**–**f** Western blotting of tumor lysates for **d** core dystroglycan (antibody AP83), **e** matriglycan-modified dystroglycan (antibody IIH6), and **f** laminin-bound dystroglycan (anti-myc, recognizing myc-tagged LG4-5 domains of laminin α1). *SkM* skeletal muscle. PCB69, eRMS 1.7-yo male. PCB178, eRMS 3-yo male. PCB42, aRMS 9-yo male (fusion unknown). PCB481, aRMS 14-yo male (Pax3:Foxo1+). CF1, aRMS 2-yo male (Pax3:Foxo1). CF2, 7-yo female (Pax7:Foxo1). PCB117, rhabdomyoma 57-yo female

The first sample-set tested was the Children’s Oncology Group Lookback tissue microarray (P1542), which includes both aRMS and eRMS subtypes. Nearly all eRMS and aRMS samples that were examined were negative for IIH6 staining, whereas normal skeletal muscle was positive (Fig. 1a–c). To determine whether the lack of matriglycan immunoreactivity in rhabdomyosarcomas reflects the loss of the core α-dystroglycan protein or a defect in its processing, we next analyzed RMS tumors by immunoblotting. In all of the samples tested, bands representing both α-dystroglycan (120–140 kDa) and β-dystroglycan (~ 43 kDa) were present (Fig. 1d). However, the two eRMS samples, as well as one of the aRMS samples, showed loss of both matriglycan immunoreactivity (Fig. 1e) and laminin binding (Fig. 1f). These results demonstrate that although the expression of dystroglycan is generally maintained in RMS tumors, the mature form of the protein is not always present.

**Fig. 2:**
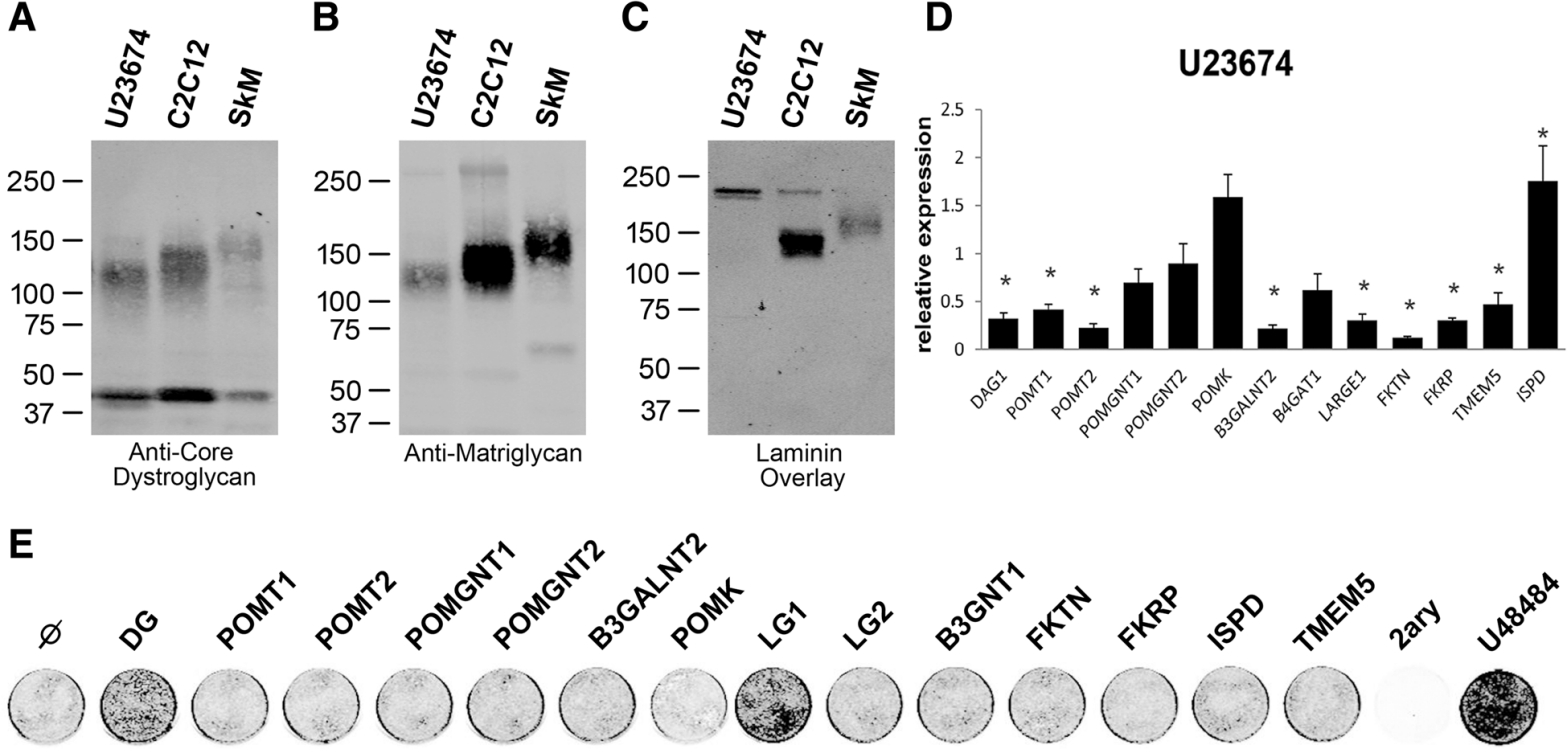
Matriglycan in the mouse RMS-derived cell line U23674. **a**–**c** Western blotting of WGA-enriched protein samples from the U23674 or C2C12 cell line, and from skeletal muscle (SkM), for core α-dystroglycan (**a**), matriglycan-modified α-dystroglycan (**b**), and laminin-bound dystroglycan (**c**). **d** Relative expression of mRNAs encoding dystroglycan (DAG1) and the dystroglycan-modifying glycosyltransferases in U23674 vs. C2C12 cells, as quantified by real-time PCR. The 12 genes tested are those known to be directly or indirectly involved in its functional glycosylation. *Rpl27* was used as the normalizing control. U23674 gene expression was normalized to C2C12. * These values are statistically different according to unpaired two-sample *t* test, *p* < 0.05. **e** Matriglycan levels in U23674 cells infected with adeno-viral vectors expressing the dystroglycan-modifying glycosyltransferases, as assessed by OnCell Western assay using an anti-matriglycan antibody. The U48484 cell line serves as a positive control

We next sought to develop a cell culture model for biochemical and expression studies. We first tested a mouse aRMS cell line (U23674) for functional glycosylation of α-dystroglycan and the expression of various enzymes involved in this process. We found that although the amounts of α-dystroglycan and β-dystroglycan in this line were similar to those in the C2C12 normal muscle cell line and in mouse skeletal muscle (Fig. 2a), matriglycan immunoreactivity and laminin binding were lower than in both controls (Fig. 2b, c). Notably, comparing the expression of genes encoding glycosyltransferases that are known to modify α-dystroglycan in the U23674 vs C2C12 line did not reveal any changes large enough to explain the observed reduction in matriglycan immunoreactivity (Fig. 2d); based on our experience assessing changes in the expression of such genes in cell culture, only a reduction to 20% or less results in measurable α-dystroglycan hypoglycosylation. Since we could not rule out the existence of inactivating mutations in any of these genes, we further tested U23674 cells for functional complementation by overexpressing individual glycosyltransferases. For this purpose, we infected these cells with adeno-viral vectors coding for each of the known α-dystroglycan-modifying glycosyltransferases (individually) and tested the glycosylation status of α-dystroglycan using the anti-matriglycan antibody. Of these, *LARGE1* was the only gene tested whose expression was able to restore matriglycan immunoreactivity (Fig. 2e).

**Fig. 3:**
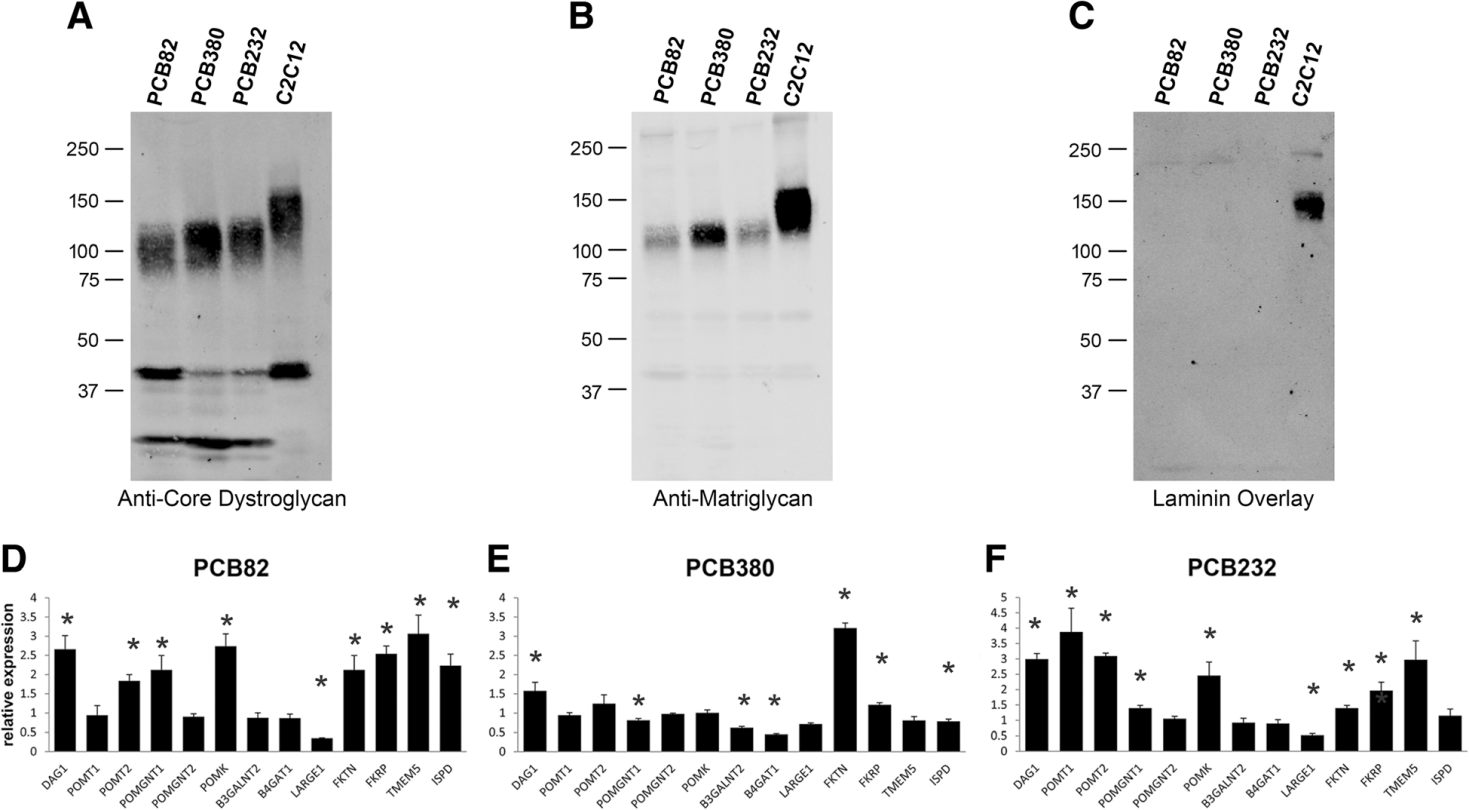
Dystroglycan glycosylation in primary cell cultures derived from human rhabdomyosarcomas. **a**–**c**. Western blotting of WGA-enriched protein samples extracted from human aRMS (PCB82 and PCB232), eRMS (PCB380), and C2C12 cells for core α-dystroglycan (**a**), matriglycan-modified α-dystroglycan (**b**), and laminin-bound dystroglycan (**c**). **d**–**f** Relative expression of mRNAs encoding dystroglycan (DAG1) and the dystroglycan-modifying glycosyltransferases in two aRMS and one eRMS line vs. skeletal muscle, as quantified by real-time PCR. * These values are statistically different according to unpaired two-sample *t* test, *p* < 0.05

We then analyzed two cell lines obtained from human eRMS tumors (PCB82 and PCB232) and one from an aRMS tumor (PCB380). All three cell lines produced bands that were detected by the anti-core dystroglycan antibody (Fig. 3a). However, matriglycan immunoreactivity was low compared to that in C2C12 muscle cells (Fig. 3b) and laminin binding was nearly eliminated (Fig. 3c). The latter defect was especially pronounced in the two eRMS cell lines, PCB82 and PCB232. However, when RT-PCR analysis was used to compare expression of the glycosyltransferases in these three cell lines, no decreases in expression were sufficient to explain the reduced matriglycan immunoreactivity and laminin binding that were detected (Fig. 3d). Thus, unidentified factors seem to underlie the loss of α-dystroglycan function in these cell lines.

**Fig. 4:**
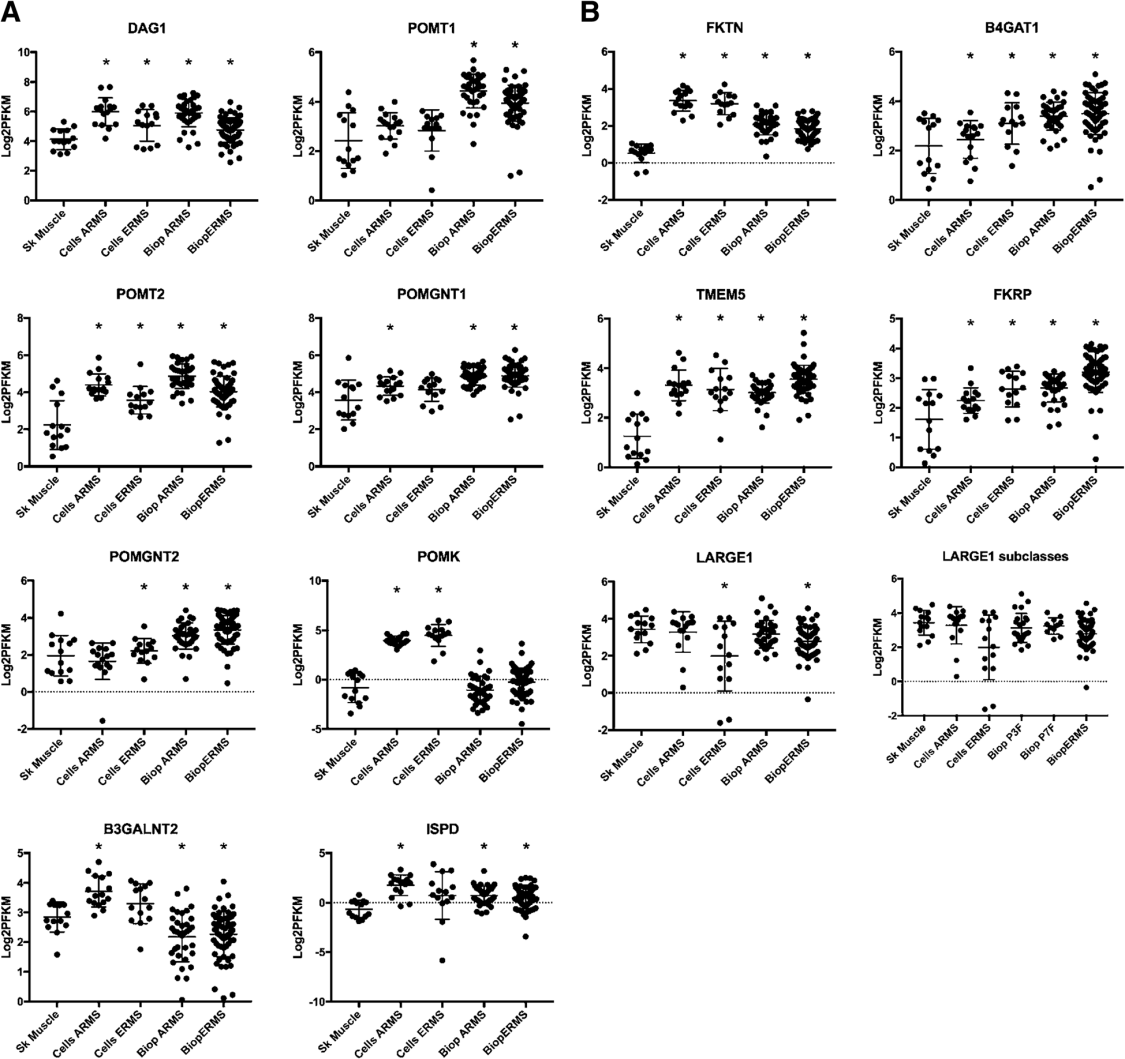
Expression of mRNAs encoding dystroglycan-modifying enzymes in human RMS. **a**, **b** Expression of the genes that code for the dystroglycan-modifying enzymes in RMS biopsies and RMS-derived cell lines relative to skeletal muscle (Sk muscle), based on publicly available RNAseq data (phs000720.v2). Each dot represents levels of the indicated mRNA from a single tumor, cell line, or skeletal muscle. Cells, cell cultures. Biop, patient biopsy. * These values are statistically different according to Mann-Whitney *U* test, *p* < 0.05

**Fig. 5:**
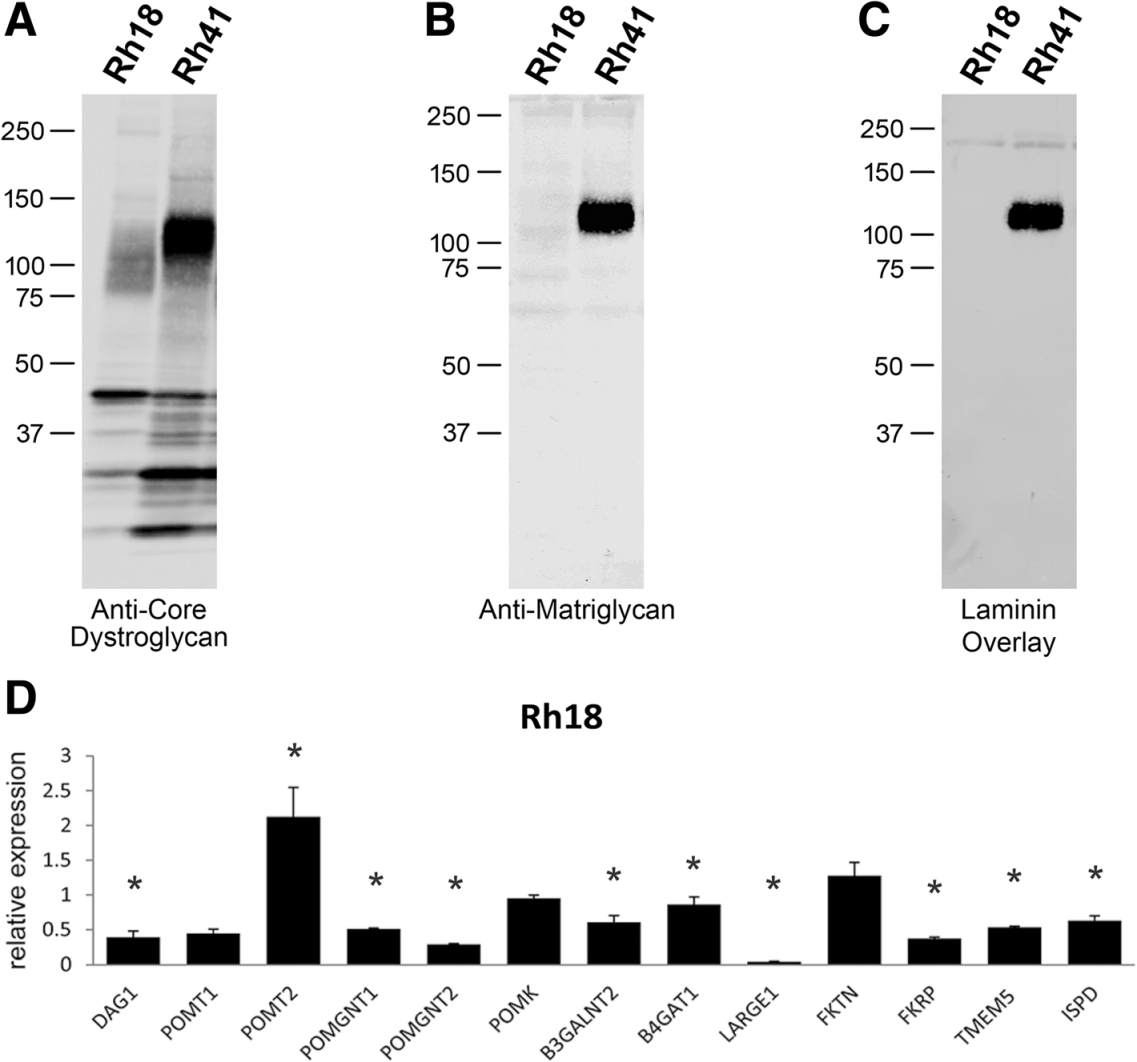
Matriglycan levels in human eRMS and aRMS cell lines. **a**–**c** Western blotting of WGA-enriched protein samples extracted from the human eRMS cell line Rh18 and the aRMS cell line Rh41 for core α-dystroglycan (**a**), matriglycan-modified α-dystroglycan (**b**), and laminin-bound dystroglycan (**c**). **d** Relative expression of mRNAs encoding dystroglycan (DAG1) and the dystroglycan-modifying glycosyltransferases in the Rh18 cell line, as assessed by real-time PCR. * These values are statistically different according to unpaired two-sample *t* test, *p* < 0.05

We next interrogated a publicly available RNAseq dataset of RMS biopsies and cell lines [26] for expression of the genes known to be involved in the functional modification of α-dystroglycan (Fig. 4a, b). Only two genes were downregulated at statistically significant levels in the RMS biopsy samples: *B3GALNT2* and *LARGE1*. Whereas *B3GALNT2* was downregulated in both ARMS and ERMS biopsies but not the corresponding cell lines, *LARGE1* was downregulated specifically in the ERMS cells and biopsy samples. For further studies, we selected two highly utilized human RMS cell lines from this panel: Rh18 (eRMS), which expresses low levels of *LARGE1*; and Rh41 (aRMS), which expresses *LARGE1* at normal levels. Analysis of both cell lines revealed that Rh18 is nearly devoid of functional α-dystroglycan as reflected by a lack of matriglycan and by a loss of activity in the laminin overlay assay (Fig. 5a–c). Notably, these defects were accompanied by a nearly complete loss of *LARGE1* expression (Fig. 5d).

**Fig. 6:**
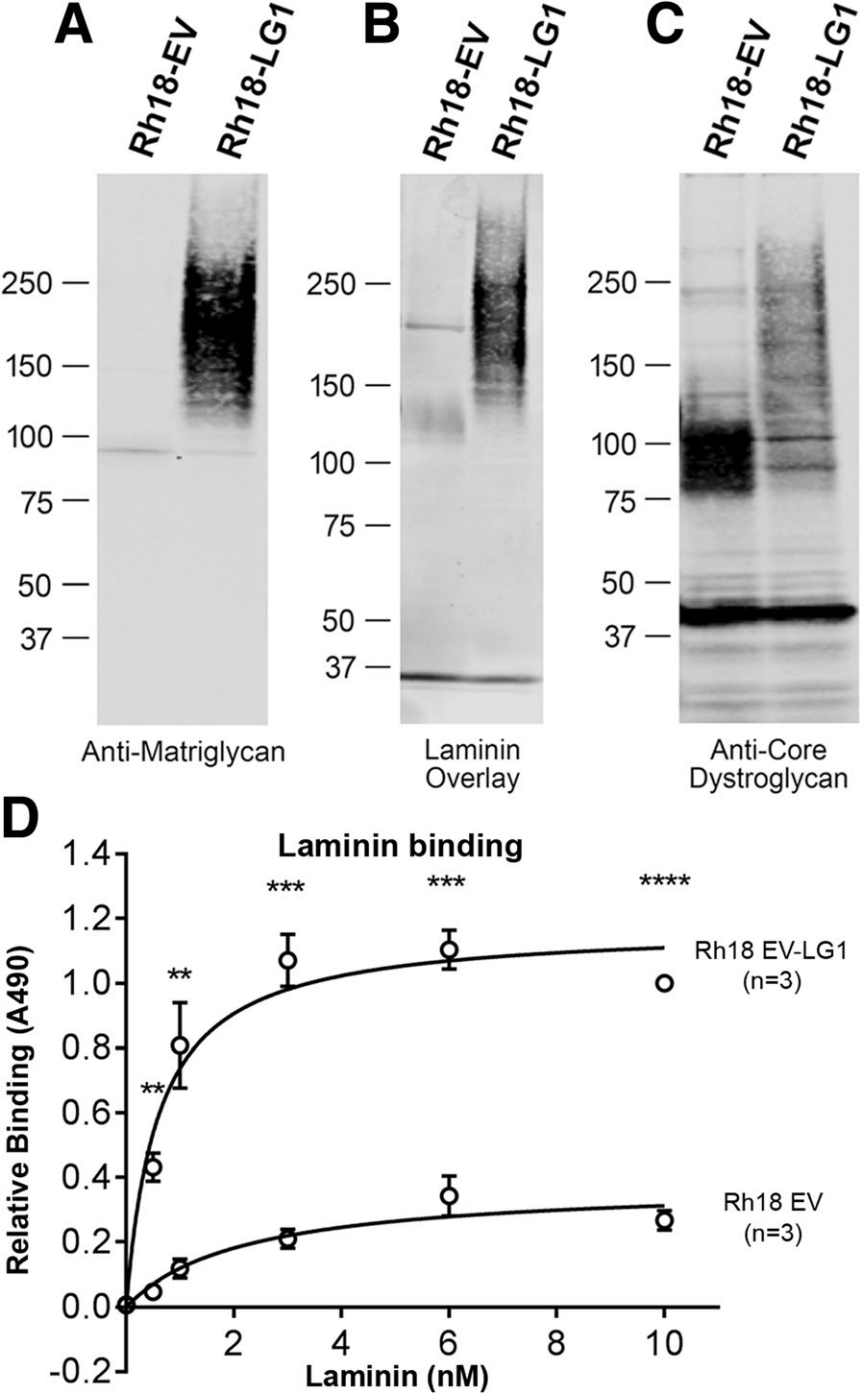
Effects of *LARGE1* expression in Rh18 cells. **a**–**c** Western blotting of the parent cell line (Rh18) and Rh18 cells transduced with particles produced by an adenovirus encoding LARGE1 for matriglycan-modified α-dystroglycan (**a**), laminin-bound dystroglycan (**b**), and core α-dystroglycan (**c**). **d** Laminin binding to Rh18 cells and Rh18 cells infected with adenovirus-expressing LARGE1. Error bars, s.e.m.; *n* = 3, experimental replicate; **p < 0.01; ***p < 0.001; ****p < 0.0001

Lastly, we tested whether ectopic expression of recombinant *LARGE1* in Rh18 cells is sufficient to restore the modification of α-dystroglycan with matriglycan. To this end, we transduced Rh18 cells with particles produced by an adeno-viral vector encoding LARGE1 (Ad-LG1) and compared the resulting cells to the parental (non-transduced) line (Fig. 6). We found that Rh18-LG1 cells produced a form of α-dystroglycan whose MW (150–200 kDa) (Fig. 6a) is similar to that in muscle and that *LARGE1* overexpression restored both the addition of matriglycan onto α-dystroglycan (Fig. 6a) and *significantly restored* its high-affinity laminin-binding function (Fig. 6b, d).

## Discussion

The DGC plays a well-established role in the formation, function, and regeneration of skeletal muscle fibers. The importance of this complex is highlighted by the muscle pathology observed in individuals with germline mutations in the genes encoding components of this complex and the glycosyltransferases involved in the modification of α-dystroglycan. Based on studies performed in both animal models and tumor samples, the DGC has also been suggested to contribute to the development of RMS [17, 18].

The first indication that the DGC might play a role in RMS was the high frequency of this type of tumor in mice deficient for α-sarcoglycan and dystrophin, two central components of the DGC. In addition, a study of dystroglycan in pediatric cancers showed that α-dystroglycan is misprocessed in a subset of human patients with RMS, although the exact mechanism was not determined [19]. In the current study, we have expanded on these findings by analyzing tumor samples and cell lines derived from mouse and human RMS. We show that both eRMS and aRMS tumors retain expression of the α-dystroglycan and β-dystroglycan subunits, but that α-dystroglycan is not modified with matriglycan, the heteropolysaccharide that confers its ligand-binding ability. Given that abnormal glycosylation has been observed in many other cancer types and is correlated with a poor prognosis [12–15], we extended this study to a series of cell lines derived from mouse and human RMS tumors. This revealed that the reduction in matriglycan levels is a common feature in cell lines derived from both mouse and human RMS.

In epithelium-derived cancers, downregulation of glycosyltransferase expression has been found to underlie abnormal glycosylation of α-dystroglycan [14, 15, 27]. Strikingly, although a reduction in *LARGE1* expression could account for the observed loss of matriglycan in particular, in certain RMS cases the collective decrease in the expression of several genes coding for α-dystroglycan-modifying glycosyltransferases is substantial, although this decrease varies across tumor types. This heterogeneity is consistent with other factors affecting the functional glycosylation of α-dystroglycan in RMS. On the other hand, ectopic expression of *LARGE1* in cell lines that are characterized by a lack of matriglycan modification on α-dystroglycan can restore both the modification (similar to levels in cell lines derived from skeletal muscle) and the ability of α-dystroglycan to function as a laminin receptor.

We previously reported that *LARGE1* is repressed in many epithelial cancer-derived cell lines, and showed that its ectopic expression reduces the invasiveness of such cells [13]. LARGE1 synthesizes and transfers repeating units of [-GlcA-beta1,3-Xyl-alpha1,3-]_n_ onto α-dystroglycan [28], forming the terminal glycan moiety anchored by the Core M3 structure on α-dystroglycan. This structure resembles the ligand-binding glycan and its length correlates with the affinity of α-dystroglycan for its ligands [25]. The fact that ectopic expression of *LARGE1* restores the matriglycan modification in all of the RMS cell lines tested, irrespective of the internal levels of *LARGE1*, indicates that LARGE 1 restructures the binding of α-dystroglycan so that it functions as an ECM receptor in RMS. Correspondingly, in the RMS cell line Rh18, ectopic expression of LARGE1 resulted in functional glycosylation of α-dystroglycan and conferred its ability to bind laminin, its natural ligand, with high-affinity.

With respect to whether defects in the glycosylation of α-dystroglycan might contribute to tumor initiation versus progression, it seems more likely that it is a secondary (somatic) event involved in tumor progression. After the metastatic cancer cell has reached its secondary site, it may restore *LARGE1* expression in order to orchestrate the remodeling of the ECM in the growing secondary tumor. Although germline mutations in *LARGE1* have been reported in a group of congenital muscular dystrophies that also involve brain and eye defects, no increase in the rate of any cancer type, including RMS, has been described in patients. However, these are rare diseases and the number of patients available for such studies is limited. Similarly, higher cancer rates have not been reported in patients who carry mutations in dystrophin or α-sarcoglycan, two other components of the DGC that have been linked to RMS [18]. We reported that ectopic expression of *LARGE1* in these cell lines enables cells to bind laminin and reduces the invasiveness of the phenotype. Recently, two other dystroglycan-modifying glycosyltransferases, LARGE2 and B4GALNT2, were linked with the cancer-associated abnormal glycosylation of α-dystroglycan [14, 15]. Although the exact mechanisms underlying this phenotype are unknown, silencing of glycosyltransferase expression could play a role in some of these cancers. The current study is focused on establishing a proof of principle that LARGE1-driven loss of matriglycan is a common feature in rhabdomyosarcomas. Future studies will not only address the effect that matriglycan restoration may have on cell attachment and differentiation but also on in vivo invasion and metastasis.

## Conclusions

We have demonstrated that the modification of α-dystroglycan with matriglycan is dramatically reduced in a panel of RMS samples, and that this result is reproduced in RMS-derived cell lines. In some of the cell lines studied, the loss of matriglycan modifications could be attributed to the silencing of *LARGE1* expression. This defect was reverted by ectopic expression of *LARGE1* cDNA in all of the cell lines that were tested, which then showed normal levels of matriglycan modifications and restored the ability of α-dystroglycan to bind to laminin. Thus, ectopic expression of *LARGE1* may be worthy of further investigation as a potential therapeutic approach for RMS.

## Abbreviations

aRMS: Alveolar rhabdomyosarcoma
CK: Creatine kinase
DGC: Dystrophin-glycoprotein complex
DMD: Duchenne muscular dystrophy
ECM: Extracellular matrix
eRMS: Embryonal rhabdomyosarcoma
LARGE1: Like-acetylglucosaminyltransferase
RMS: Rhabdomyosarcoma
